# Reliable Identification of Deep Sulcal Pits: The Effects of Scan Session, Scanner, and Surface Extraction Tool

**DOI:** 10.1371/journal.pone.0053678

**Published:** 2013-01-07

**Authors:** Kiho Im, Jong-Min Lee, Seun Jeon, Jong-Heon Kim, Sang Won Seo, Duk L. Na, P. Ellen Grant

**Affiliations:** 1 Division of Newborn Medicine, Boston Children's Hospital, Harvard Medical School, Boston, Massachusetts, United States of America; 2 Center for Fetal Neonatal Neuroimaging and Developmental Science, Boston Children's Hospital, Harvard Medical School, Boston, Massachusetts, United States of America; 3 Department of Biomedical Engineering, Hanyang University, Seoul, Korea; 4 Department of Neurology, Samsung Medical Center, Sungkyunkwan University School of Medicine, Seoul, Korea; 5 Department of Radiology, Boston Children's Hospital, Harvard Medical School, Boston, Massachusetts, United States of America; Beijing Normal University, China

## Abstract

Sulcal pit analysis has been providing novel insights into brain function and development. The purpose of this study was to evaluate the reliability of sulcal pit extraction with respect to the effects of scan session, scanner, and surface extraction tool. Five subjects were scanned 4 times at 3 MRI centers and other 5 subjects were scanned 3 times at 2 MRI centers, including 1 test-retest session. Sulcal pits were extracted on the white matter surfaces reconstructed with both Montreal Neurological Institute and Freesurfer pipelines. We estimated similarity of the presence of sulcal pits having a maximum value of 1 and their spatial difference within the same subject. The tests showed high similarity of the sulcal pit presence and low spatial difference. The similarity was more than 0.90 and the spatial difference was less than 1.7 mm in most cases according to different scan sessions or scanners, and more than 0.85 and about 2.0 mm across surface extraction tools. The reliability of sulcal pit extraction was more affected by the image processing-related factors than the scan session or scanner factors. Moreover, the similarity of sulcal pit distribution appeared to be largely influenced by the presence or absence of the sulcal pits on the shallow and small folds. We suggest that our sulcal pit extraction from MRI is highly reliable and could be useful for clinical applications as an imaging biomarker.

## Introduction

The first sulci form while radial migration of neurons forms the cerebral cortex. Early gyrogenesis has been hypothesized to be more influenced by genetic than environmental factors because of the relative invariant spatial distribution of early sulci, which may be due to a human-specific predetermined protomap of functional areas [1], [2], [3], [4], [5]. It has been suggested using functional magnetic resonance imaging (MRI) that early-developing sulci are predictive for functional area locations and spatially covary with them [6], [7], [8]. It is therefore important to identify those first folds and analyze them with functional features for understanding the anatomical and functional development of the human brain.

The first folds can be estimated in mature brains because they are thought to develop into the deepest local regions of sulci, called the ***sulcal pits***. We have extracted and analyzed sulcal pits from MRI data and supported the hypothesis about their biological meaning. We have analyzed the spatial distribution of sulcal pits on the cortical surface and have found hemispherical asymmetry in the superior temporal regions, which may be related to the left lateralization of language function [2]. We have also reported the significant relationship between the presence of sulcal pits and intellectual ability estimated with intelligence quotient (IQ) scores [9]. In the high verbal IQ group a sulcal pit was more frequently present in the left posterior inferior frontal sulcus (near Broca's area) and the right posterior inferior temporal sulcus, regions reported to be involved in language function [10]. Other recent studies have presented a graph-based sulcal pattern comparison method using sulcal pits and applied this method to twins and polymicrogyria patients [11], [12]. We showed that the similarity of the 3D position of the sulcal pits in twin pairs was strongly higher than in unrelated pairs, supporting the hypothesis that the spatial distribution of sulcal pits might be under tight genetic control [11]. Our previous studies suggest that sulcal pits have the potential to be a new important neuroimaging biomarker of brain function and development.

Methodologically, a sulcal pit is defined as the point having a local maximum depth on the inner cortical surface (white matter surface) [2] and therefore the extraction of sulcal pits is affected by the gray/white matter surface reconstruction. Gray/white surface reconstruction is in turn affected by image characteristics such as gray/white matter intensity and signal to noise and image processing. MR intensity properties can be influenced by subject-related factors, such as hydration status [13] or blood pressure and instrument-related factors such as field strength, scanner hardware, imaging magnetic gradients or pulse sequence [14], [15], [16], [17], [18]. Different image processing such as software package or the parameters chosen for the processing, when the identical image is used may also contribute to variability in sulcal pit extraction. For example, the cortical surface reconstruction algorithms used in the Montreal Neurological Institute (MNI) [19], [20], Freesurfer (FS) [21], [22], and BrainVISA [23] pipelines result in different geometric accuracy and different surface mesh characteristics [24]. Therefore, these factors may affect surface reconstructions and sulcal pit extraction. Although we have shown that our method detected appropriate sulcal pits and their spatial distribution across subjects [2], intra-subject reliability and reproducibility has not been evaluated. It is important to determine the reliability of sulcal pit extraction under different scanning conditions and with different image processing factors to determine if sulcal pits have a potential role as an imaging biomarker.

Therefore the purpose of this study was to investigate the effects of scan session, scanner, and surface extraction tool on the reliability of the sulcal pit extraction. Healthy young subjects were scanned several times on different scanners and their images were processed to extract sulcal pits on the cortical surface. We reconstructed cortical surfaces with 2 different surface extraction pipelines, the MNI [19], [20] and FS [21], [22] pipelines, which have been largely used for cortical thickness and shape analyses. Sulcal pit maps acquired from different images, or different surface extraction pipelines were compared to each other within the same subject by measuring the similarity of the presence of sulcal pits and their spatial difference.

## Materials and Methods

### Data acquisition

Seven male and 3 female healthy volunteers (age, mean ± standard deviation [SD]: 26.1±2.9 years) were recruited who had no historical background of psychological illness or neurological disorder. Five subjects were scanned 4 times at 3 MRI centers (Samsung Seoul Medical Center [SMC], Ewha-woman's University Mokdong Medical Center [EUMC], and Asan Medical Center [AMC]), and the other 5 subjects were scanned 3 times at 2 centers (SMC and EUMC) including 1 test-retest session at SMC (SMC_1_ and SMC_2_). Two sessions of test-retest were approximately 3 weeks apart and all other sessions were within this period. As in previous studies of MRI-based morphometric structural reliability [15], [16], 3-week intervals would include the source of variability relevant to subject-related factor, such as hydration status or blood pressure, and instrument-related factors, such as scanner drift, which may be minimized when the test-retest interval is several minutes to ∼1 day. We obtained written consents from each patient and the Institutional Review Board of the Samsung Medical Center, Ewha-woman's University Mokdong Medical Center and Asan Medical Center approved the study protocol.

Four or 3 sets of MRI data were acquired on the scanners of same MR manufacturer using same sequence (Philips 3T Achieva scanners, T1-weighted 3D-TFE (Turbo Echo Field) sequence). The 3 MRI centers used identical parameters for isotropic 0.5 mm acquisition: sagittal slice thickness  = 1.0 mm, overcontiguous slices with 50% overlap, no gap, TR  = 9.9 ms, TI  = 1,245 ms, TE  = 4.60 ms, Bandwidth  = 142.3 Hz/pixel, flip angle  = 8°, matrix size of 240×240 pixels, reconstructed to 480×480 over a FOV of 240 mm, voxel size  = 0.5×0.5×0.5 (mm). High resolution structural image is sensitive to head motion even within the single volume. We inspected all raw images and assured that there was no head motion.

### Image processing and cortical surface extraction using the MNI and FS pipelines

In the MNI pipeline, the native images were normalized to a standardized stereotaxic space using a linear transformation and corrected for intensity nonuniformity [25], [26]. Images were, then, classified into white matter, gray matter, cerebrospinal fluid, and background using an advanced neural net classifier [27]. The hemispherical surfaces of the gray/white matter boundary and gray matter/cerebrospinal fluid boundary) were automatically extracted, consisting of 40,962 vertices [19], [20].

The FS pipeline includes removal of non-brain tissue [28], stereotaxic space transformation, tissue segmentation, intensity normalization [25], tessellation of the gray/white matter boundary, automated topology correction [29], [30], and surface deformation following intensity gradients to optimally place the gray/white matter and gray matter/cerebrospinal fluid boundaries [21].

### Extraction of sulcal pits on the cortical surface

A sulcal pit is the deepest local point in a sulcal catchment basin, and can be identified by using a sulcal depth map on the cortical surface. We used the white matter surface (gray/white matter boundary) and the 3D Euclidean depth map to extract sulcal pits [2], [9]. The 3D Euclidean sulcal depth maps were generated by measuring the Euclidean distance from each vertex in the cortical surface to the nearest voxel on the cerebral hull [2], [31]. We used a watershed algorithm based on a depth map to extract sulcal pits on triangular meshes. To prevent overextraction of the pits, we first reduced noisy depth variations by surface-based heat kernel smoothing with a full-width half-maximum value of 10 mm [32]. Subsequently we performed segment merging in the watershed algorithm using the area of the catchment basin, the distance between the sulcal pits, and the ridge height. If one of the areas of two or more catchment basins was smaller than a threshold (30 mm^2^) when they met at a ridge point, the smaller catchment basin below the threshold was merged into the adjacent catchment basin with the deepest pit and its sulcal pit removed. If the distance between two pits was less than a 15 mm threshold, the shallower pit was also merged into the deeper one. Finally, merging was executed when the ridge was lower than a threshold of 2.5 mm. The methodological procedure was explained in more detail in our previous study [2].

### Intra-subject cortical surface alignment

**Figure 1:**
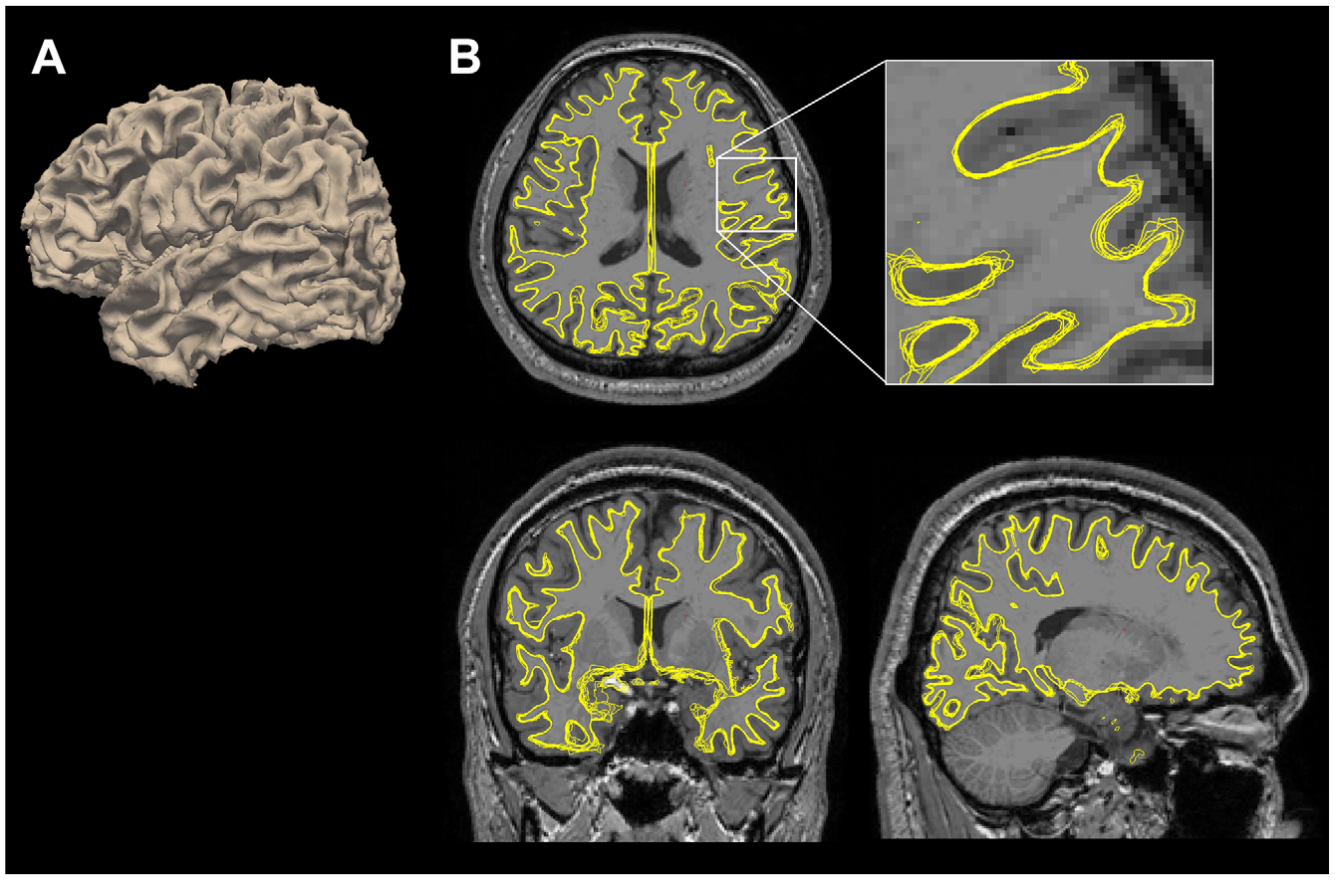
Example of intra-subject cortical surface alignment. Eight white matter surfaces (4 MNI and 4 FS surfaces) are rendered (A) and they are overlapped with a reference volume image (B) after a linear registration with 6 parameters. They are aligned well into a common space, but their local shape of the gray/white matter boundary is slightly different.

Surface vertex correspondence must be built to compare the results of sulcal pit extraction. In the MNI and FS pipelines, corresponding regions between subjects are determined using their own surface-based registration methods with a sphere-to-sphere matching in which the vertices of each subject are nonlinearly registered to a template surface [33], [34], [35]. However, our reliability test needs not inter- but intra-subject comparison. The nonlinear registration between surfaces of the same subject could give rise to extra noise in the correspondence definition. In addition, when using any specific surface registration algorithm, its performance and accuracy could be biased by different mesh properties of the MNI and FS surfaces. Instead, we adopted a volume linear registration to perform the intra-subject surface alignment [15]. We randomly chose 1 reference target volume for each subject and the other 3 or 2 volumes were registered to the target volume using the MNI linear registration tool with 6 parameters. The transformation matrix of the registration was then applied to the corresponding surface. Finally, 8 or 6 surfaces from each individual subject (MNI and FS surfaces from 4 or 3 images) were registered to a common space. In order to check the registration result, we overlapped all surfaces to a reference volume image in the same space (Fig. 1). It is confirmed that their global position and orientation are matched and the linear registration is sufficient and robust for intra-subject surface alignment. Alignment results of 8 surfaces for the other 4 subjects are provided in supplementary data (Fig. S1). However, since the surfaces were reconstructed from different images or tools, the local shape of the gray/white matter boundary was slightly different as shown in Fig. 1. It would lead to differences in the presence and location of sulcal pits.

### Evaluation of reliability for sulcal pit extraction

**Figure 2:**
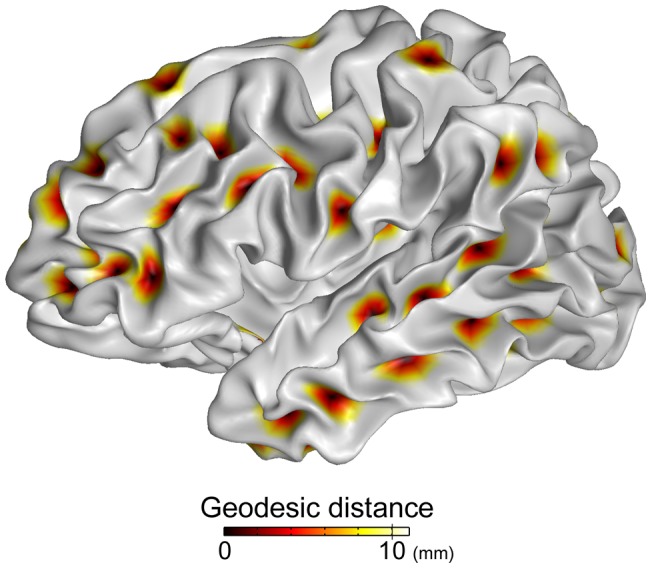
Geodesic distance map from sulcal pits as a seed point on the white matter surface. The areas within 10 mm distance from the sulcal pits are separated from each other.

After the global surface alignment on the common space, we compared the map of sulcal pits between different surfaces of the same subject across the entire cortex. Because of different surface shape on the gray/white matter boundary, sulcal depth maps were not identical with each other. The following process including the surface-based smoothing and the merging in the watershed algorithm might also cause inconsistent identification of the local maximum depth points. First, our reliability test was to observe the variability of the presence of sulcal pits. Second, if two pits existed in the same anatomical region, we measured how much their locations are different. Given a pair of surfaces, *S_A_* and *S_B_*, containing sulcal pits *P_A_*  =  {*a_1_*, *a_2_*, …} and *P_B_*  =  {*b_1_*, *b_2_*, …} respectively, we estimated similarity of the presence of sulcal pits and their spatial difference by detecting the sulcal pits which exist in the same sulcal catchment basin. We assumed that if two pits from *P_A_* and *P_B_* are close to each other and so their spatial difference is less than a threshold of 10 mm, they are matched and identified in the same catchment basin. Matching of two pits from different sulcal basins might be expected, but there is low probability for that case. As described above, noisy sulcal pits that were extracted from small catchment basins and those that were located too close to other pits were merged and removed, therefore by construction the area within 10 mm distance from the sulcal pit did not encroach into other sulcal pit areas. Fig. 2 shows the map of geodesic distance from sulcal pits as a seed point in an individual cortical surface for an example. It is shown that areas within 10 mm distance from the sulcal pits are separated from each other.

**Figure 3:**
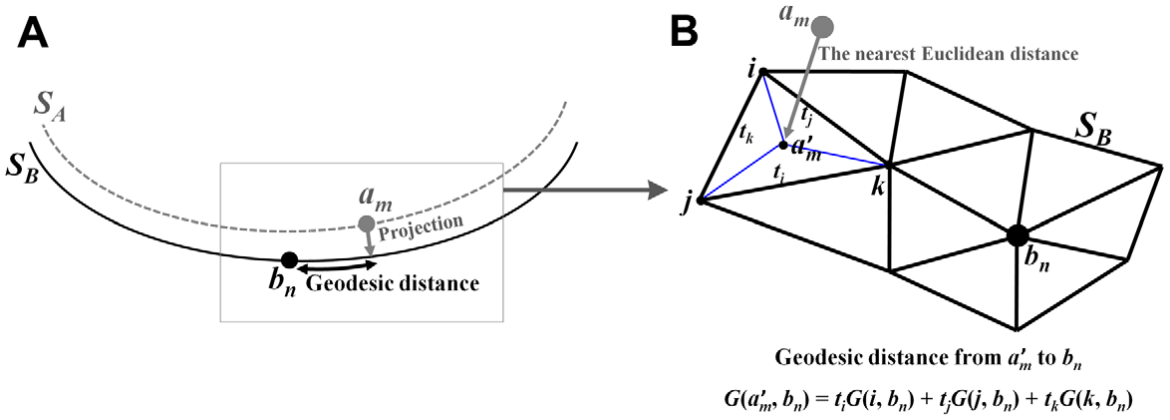
Schematic illustration of measuring spatial difference between 2 pits from different surfaces. Sulcal pit *a_m_* on the surface *S_A_* is projected onto the surface *S_B_* with the nearest Euclidean distance (A) and then geodesic distance (*G*) between projected pit 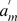
and *b_n_ G*(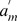, *b_n_*) is measured (B). *t_i_*, *t_j_*, and *t_k_* are the barycentric coordinates of 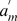 on the triangle which is composed of vertices *i*, *j*, and *k*. *G*(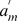, *b_n_*) is computed using an interpolation with distance values of three vertices.

We first projected the *P_A_* onto the *S_B_* with the nearest Euclidean distance and then measured the geodesic distances from the *P_B_* on the *S_B_* (Fig. 3A). The geodesic distances were computed and assigned to the vertices constituting the surface model [35], [36]. In case the nearest points from the *P_A_* to the *S_B_* were not located on the vertex but on the plane of a triangle, we performed an interpolation using known distance values of three vertices with barycentric coordinates on the triangle [37]. The schematic illustration is provided with more detail in Fig. 3B. Among the *P_A_*, we counted the number of sulcal pits matched with the *P_B_*, *N*(*P_A→B_*), whose shortest geodesic distances from the *P_B_* were less than 10 mm. At this time when the pits from different surfaces were regarded to be matched within 10 mm, we also acquired their geodesic distances and calculated the mean value *D*(*P_A→B_*). Next, the *P_B_* was projected onto the *SA* and *N*(*P_B→A_*) and *D*(*P_B→A_*) were measured with the same manner explained above. We computed the ratios of *N*(*P_A→B_*) and *N*(*P_B→A_*) to the whole number of sulcal pits *N*(*P_A_*) and *N*(*P_B_*) respectively. The similarity of the presence of sulcal pits (*M_1_*) and their spatial differences (*M_2_*) between *SA* and *SB* were finally defined as follows:
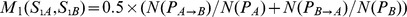


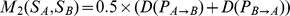
where *M_1_* and *M_2_* are symmetric matrices for the comparison of all pairs among 8 or 6 surfaces. The simple example for these measurements is shown in supplementary data (Fig. S2).

### Effects of different scan session, scanner, and surface extraction tool

**Figure 4:**
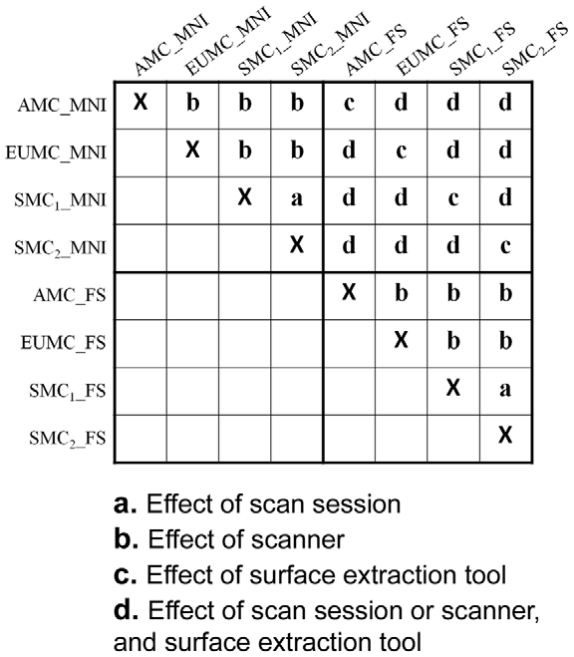
Matrix of all pairwise comparison of sulcal pit map in the same subject. The effects of different scan session, scanner, and surface extraction tool are evaluated and each case is marked in the cells of the matrix. For the subject scanned 3 times, 6×6 matrix is constructed without rows and columns of AMC_MNI and AMC_FS.

We constructed *n* × *n* matrices *M_1_* and *M_2_* (*n* = 8 or 6) and performed (*n* ^2^–*n*)/2 comparisons for each subject. Matrix of all pairwise comparison of sulcal pit maps is shown in Fig. 4. In these matrices, we evaluated the effects of different scan session, scanner, and surface extraction tool on the similarity of the presence of sulcal pits and their spatial difference. A detailed explanation of the test for 8×8 matrix is as follows:

(a) Effect of different scan session in the MNI and FS surfaces

*M_1,2_*(*S_X_MNI_*, *S*_Y*_MNI*_), *M_1,2_*(*S_X_FS_*, *S*_Y*_FS*_): *X*  =  SMC_1_ and Y  =  SMC_2_

Sulcal pit maps were compared in the pair of repeated scans from the same SMC scanner, which were extracted from the same surface extraction tool.

(b) Effect of different scanner in the MNI and FS surfaces

*M_1,2_*(*S_X_MNI_*, *S*_Y*_MNI*_), *M_1,2_*(*S_X_FS_*, *S*_Y*_FS*_): *X* ≠ Y and (*X* ≠ SMC or Y ≠ SMC)

We compared the sulcal pit maps of the pair extracted from the same surface extraction tool but different scanner. There were 5 possible pairs to be compared for the MNI and FS surfaces respectively.

(c) Effect of different surface extraction tool

*M_1,2_*(*S_X_MNI_*, *S*_Y*_FS*_): *X* = Y

The difference of sulcal pit maps was measured between the MNI and FS surfaces in the 4 pairs who came under the same scan session and scanner.

(d) Effects of different scan session or scanner, and surface extraction tool

*M_1,2_*(*S_X_MNI_*, *S*_Y*_FS*_): *X*≠Y

The sulcal pit maps of all other 12 pairs acquired from different scan session or scanner, and surface extraction tool were compared.

As shown in Fig. 4, each case is marked in the cells of the matrix. For the cases of b, c, and d, there were several comparisons, so we measured the mean values respectively.

### Sulcal depth and area of sulcal catchment basin on matched and unmatched sulcal pits

We performed the supplementary analysis to find the structural characteristics of the regions where sulcal pit presence was not consistent. In the process of the similarity measure of sulcal pit presence, matched and unmatched sulcal pits from the pair of 2 surfaces were differentiated. Measuring the depths and areas of the sulcal basin of unmatched sulcal pits can provide morphological information on the regions showing variable sulcal pit presence. We computed the means of these measurements in each subject and compared them with the same measurements of matched sulcal pits. The differences between matched and unmatched sulcal pits were examined with a paired *t*-test.

## Results

**Figure 5:**
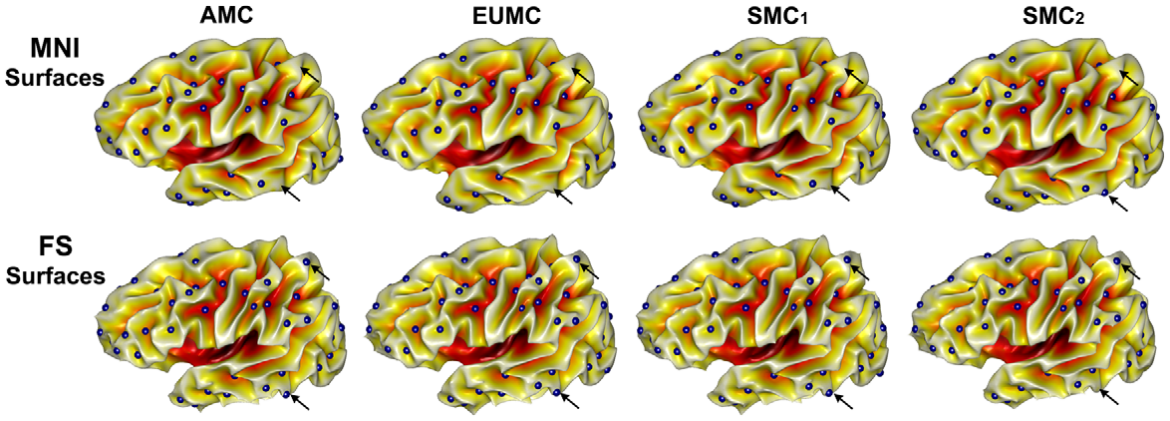
Sulcal pit maps on the different 8 surfaces in the same subject. The location and existence of the sulcal pits are highly similar across surfaces. All surfaces are inflated for better visualization. The regions marked with black arrows show the variability of the sulcal pit presence.

**Figure 6:**
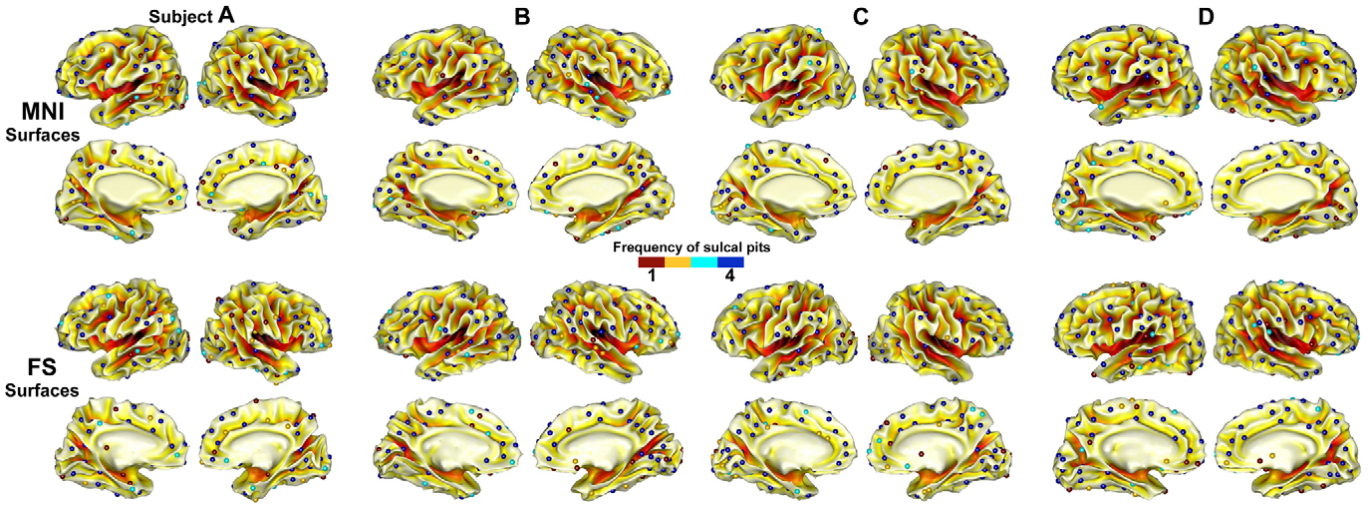
Sulcal pit map for each subject representing the frequency of sulcal pits across the 4 MNI and 4 FS surfaces. All sulcal pits from different surfaces are projected onto one surface, and the frequency of sulcal pits overlaid in the same region is mapped with color.

We display the sulcal pit maps from different 8 surfaces for an individual brain. When we visually compare the location and existence of the sulcal pits, their distributions are highly similar to each other although the presence of sulcal pits is variable in some regions as marked in Fig. 5. We also projected all sulcal pits onto one cortical surface and constructed a pit representation for each subject indicating the frequency of sulcal pit presence across the 4 MNI and 4 FS surfaces (Fig. 6). The same representation is provided for the other 5 subjects scanned 3 times in Fig. S3. Our quantitative reliability evaluation shows the similarities of the presence of sulcal pits and their spatial differences in both hemispheres for each subject with respect to different scan session, scanner, and surface extraction tool, which are presented in Table 1 and 2. The test-retest comparisons from the same scanner showed the mean similarities of sulcal pit presence from 0.905 to 0.920 in both MNI and FS surfaces (left MNI [mean]: 0.907, right MNI: 0.906, left FS: 0.917, right FS: 0.920). In the measurement of spatial differences between matched pits, the mean differences were less than 1.7 mm (left MNI: 1.66 (mm), right MNI: 1.68, left FS: 1.59, right FS: 1.62). When comparing the sulcal pit presence across different scanners with the same surface extraction tool, the mean similarities were about 0.905 in both hemispheres (left MNI: 0.904, right MNI: 0.901, left FS: 0.918, right FS: 0.908). Their corresponding mean spatial differences were less than 1.8 and 1.7 mm in the MNI and FS surfaces respectively (left MNI: 1.75, right MNI: 1.78, left FS: 1.55, right FS: 1.62). The test of the effect of surface extraction tools showed that the mean similarities of the presence of sulcal pits were 0.852 and their mean spatial differences were 1.92 and 2.05 mm for left and right hemispheres. When scan session, scanner, and surface extraction tool were all different, the mean similarities of sulcal pit presence in the left and right hemispheres were 0.856 and 0.850, and the mean spatial differences were 1.95 and 2.06 mm respectively.

**Table 1:** Similarity of the presence of sulcal pits (*M_1_*) according to effects of scan session, scanner, and surface extraction tool.

Left hemisphere						
Subject ID	Scan session, MNI surface	Scan session, FS surface	Scanner, MNI surface	Scanner, FS surface	Surface extraction tool	Scan session or scanner, and surface extraction tool
1	0.884	0.924	0.883	0.918	0.831	0.839
2	0.878	0.941	0.873	0.921	0.857	0.844
3	0.923	0.907	0.923	0.913	0.856	0.852
4	0.930	0.881	0.912	0.900	0.875	0.874
5	0.922	0.906	0.916	0.911	0.854	0.857
6	0.914	0.932	0.903	0.931	0.833	0.837
7	0.895	0.888	0.902	0.898	0.863	0.862
8	0.928	0.926	0.919	0.945	0.876	0.873
9	0.910	0.920	0.905	0.900	0.844	0.859
10	0.888	0.919	0.890	0.918	0.843	0.849
Mean±SD	0.907±0.019	0.917±0.019	0.904±0.016	0.918±0.015	0.852±0.016	0.856±0.013

Right hemisphere						
1	0.904	0.881	0.908	0.880	0.828	0.828
2	0.859	0.901	0.861	0.891	0.837	0.819
3	0.922	0.933	0.926	0.923	0.886	0.885
4	0.932	0.926	0.921	0.913	0.899	0.897
5	0.895	0.912	0.880	0.890	0.833	0.842
6	0.896	0.893	0.908	0.880	0.829	0.835
7	0.885	0.938	0.912	0.922	0.856	0.857
8	0.934	0.964	0.906	0.940	0.873	0.868
9	0.913	0.900	0.889	0.895	0.843	0.838
10	0.920	0.957	0.897	0.943	0.839	0.836
Mean±SD	0.906±0.023	0.920±0.028	0.901±0.020	0.908±0.024	0.852±0.025	0.850±0.026

**Table 2:** Spatial difference (mm) of sulcal pits (*M_2_*) according to effects of scan session, scanner, and surface extraction tool.

Left hemisphere						
Subject ID	Scan session, MNI surface	Scan session, FS surface	Scanner, MNI surface	Scanner, FS surface	Surface extraction tool	Scan session or scanner, and surface extraction tool
1	1.55	1.52	1.89	1.61	1.95	2.07
2	2.01	1.91	1.82	1.61	1.82	1.87
3	1.69	1.49	1.81	1.65	1.94	1.95
4	1.56	1.83	1.6	1.47	1.79	1.79
5	1.45	1.62	1.66	1.54	2.07	1.99
6	1.48	1.55	1.75	1.51	1.92	1.95
7	1.97	1.31	1.88	1.44	1.85	1.91
8	1.57	1.45	1.72	1.45	1.78	1.94
9	1.52	1.82	1.65	1.79	2.08	2.06
10	1.81	1.44	1.70	1.46	1.98	2.01
Mean±SD	1.66±0.20	1.59±0.20	1.75±0.10	1.55±0.11	1.92±0.11	1.95±0.09

Right hemisphere						
1	1.40	1.58	1.75	1.63	1.99	1.99
2	1.82	1.50	1.94	1.61	2.18	2.21
3	2.04	1.54	1.80	1.52	1.95	2.01
4	1.55	1.68	1.66	1.58	1.97	1.97
5	1.65	1.91	1.86	2.01	2.23	2.19
6	1.83	1.74	1.92	1.64	2.39	2.39
7	1.51	1.51	1.52	1.45	1.60	1.69
8	1.75	1.69	1.89	1.70	2.18	2.11
9	1.61	1.43	1.70	1.51	2.02	2.10
10	1.60	1.66	1.76	1.52	1.95	1.94
Mean±SD	1.68±0.19	1.62±0.14	1.78±0.13	1.62±0.16	2.05±0.21	2.06±0.19

The mean depth on matched sulcal pits was more than 12 mm in both hemispheres, however the mean depth on unmatched sulcal pits was less than 10 mm. Their difference across subjects was evaluated with a paired *t*-test and was statistically significant (*P*<0.0001). The areas of sulcal catchment basin of matched sulcal pits were also significantly larger than the areas of unmatched sulcal pits in both hemispheres (*P*<0.0001). All data and statistical results are shown in Table 3.

**Table 3:** Depth and area of sulcal catchment basin measured on matched and unmatched sulcal pits and statistical tests.

Left hemisphere						
	Depth (mm)			Area of sulcal catchment basin (mm^2^)		
Subject ID	Matched pits	Unmatched pits		Matched pits	Unmatched pits	
1	12.47	10.55		1282.07	1008.81	
2	12.39	9.60		1306.76	650.79	
3	12.20	9.47		1178.82	1012.01	
4	11.88	9.42		1134.32	789.09	
5	12.43	9.07		1332.66	748.24	
6	11.36	8.83		1155.89	719.33	
7	11.32	9.05		1226.12	784.47	
8	12.78	10.36		1327.10	1032.80	
9	12.17	9.28		1239.66	884.48	
10	12.50	10.41		1331.85	725.19	
Mean±SD	12.15±0.49	9.60±0.62	*P*<0.0001	1251.53±75.78	835.52±129.31	*P*<0.0001

Right hemisphere						
1	12.78	9.87		1279.33	763.46	
2	12.83	11.28		1179.21	979.83	
3	11.97	7.83		1183.84	571.03	
4	12.90	10.80		1157.31	1069.22	
5	12.97	10.67		1223.96	659.34	
6	12.49	9.31		1278.77	702.09	
7	12.52	8.69		1208.70	667.03	
8	12.34	9.02		1324.57	705.15	
9	12.92	9.22		1338.33	673.22	
10	12.31	10.02		1227.98	890.27	
Mean±SD	12.60±0.33	9.67±1.06	*P*<0.0001	1240.20±62.31	768.07±159.39	*P*<0.0001

Paired *t*-test *P* value.

## Discussion

We acquired 4 images on 3 different scanners or 3 images on 2 scanners (1 test-retest session) from the same MR manufacturer and field strength and using the same sequence with identical parameters in 10 subjects. We constructed sulcal pit maps on the white matter surfaces, extracted with both MNI and FS pipelines, and compared with each other. We globally aligned cortical surfaces using a volume linear registration to avoid biases that might be caused by a nonlinear surface registration, and carried out pairwise comparisons by projecting sulcal pits from one surface to another one. Although there is a minute difference in the local shape between surfaces, their global positions matched well (Fig. 1 and S1). Therefore, sulcal pits can be projected to corresponding areas on another surface. The effects of scan session, scanner, and surface extraction tool were investigated for each subject.

Sulcal pit extraction from MRI was highly reliable across different scan sessions and scanners, showing high similarity of the sulcal pit presence and low spatial difference. The similarity of the presence of sulcal pits was more than 0.90 and their spatial difference was only about 1.70 mm. Typically, the edge distance between a vertex and its neighboring vertex was 1.75 to 1.85 mm and 0.83 to 0.85 mm for the MNI and FS surfaces respectively. Hence, the spatial difference of sulcal pits was quite small when considering the mesh resolution of the surface model. These results suggest that the locally deepest point in a sulcus detected from MRI is not random but is a reliable structural feature and our algorithm generates reliable sulcal pit maps on the cortical surface. In the process of sulcal pit extraction, we smoothed the depth map on the surface and performed a merging process which removes noisy pits according to several criteria and parameters. This process likely plays an effective role in increasing the reliability of sulcal pit detection. We could also have examined the reliability of sulcal pit distribution with different parameters. However, we have evaluated and optimized the parameters for sulcal pit extraction in our previous study [2], and have continuously shown significant relationships between our sulcal pit map and brain function [2], [9], [11], [12]. Thus, this study aimed at considering the reliability of sulcal pits extracted with our optimized and fixed parameters.

We also detected reliable sulcal pits with the MNI and FS image analysis tools using the same images, with the similarity of more than 0.85 and about 2.00 mm spatial difference. However, the reliability test across different tools showed lower similarity and higher spatial difference than across different scan sessions and scanners. In addition, the similarity and spatial difference didn't change significantly when both surface extraction tools and scan sessions or scanners were different. The reason the effect of surface extraction tool is higher than the effect of scan session or scanner on reliability of sulcal pit extraction may be because cortical surface extraction requires several specific procedures including intensity nonuniformity correction, intensity normalization, stereotaxic space transformation, tissue segmentation, surface modeling, and so forth. Although MNI and FS pipelines adopt the same tool for some processes, such as the N3 algorithm for the intensity nonuniformity correction [25], much of their pipelines contain different tools and algorithms. Therefore there are several different image processing steps that may have caused the variability in the presence of sulcal pits and their spatial localization. We hypothesize that a method for surface modeling might be one of the most critical steps affecting sulcal pit extraction, although further investigation is required to understand and evaluate the effect of each processing step. The MNI and FS pipelines use totally different approaches for cortical surface modeling. The white matter surface is extracted by deforming an initial spherical mesh onto the gray/white matter boundary using the Constrained Laplacian-based Automated Segmentation with Proximities algorithm in the MNI pipeline [19], [20]. In the FS pipeline, a surface tessellation is constructed by using triangles to represent face separating white matter voxels [21]. The number of triangles and the properties of surface mesh are different between the MNI and FS surfaces and their local shapes are not identical. These differences may have an impact on the generation of sulcal depth map and sulcal pit extraction reproducibility. Moreover, when we visually observe individual maps of the sulcal pit frequency and compare the maps between the MNI and FS surfaces, the presence and distribution of the sulcal pits seem more variable on the medial areas than lateral areas according to different tools (Fig. 6 and S3). Nevertheless, our results confirm that we can still expect high reliability of sulcal pit extraction from the gray/white surface. We additionally investigated differences in the number of pits between the MNI and FS surfaces with a paired *t*-test. No significant difference was found in both left and right hemispheres (left MNI [mean]: 73.8 (number of pits), left FS: 74.2, *P* = 0.746, right MNI: 75.0, right FS: 75.4, *P* = 0.617).

We analyzed the sulcal depth and area of sulcal catchment basin on matched and unmatched sulcal pits to see the structural characteristics of the regions where sulcal pit presence was not consistent. Our results showed that the sulcal depth was significantly shallower and the area of sulcal catchment basin was significantly smaller in the regions where sulcal pit presence or absence was variable according to scan sessions, scanners, or surface extraction tools. The sulcal pit presence was more consistent in the deep and large sulcal folds. These results can be confirmed in the individual representations of the sulcal pit frequency across different images (Fig. 6 and S3). Sulcal pits on the shallow and small folds are candidates for the merging in our algorithm. Sometimes they may be merged and removed, but other times they may not, due to minute changes in the modeling of cortical surface shape. However, once sulcal pits are identified, they showed invariant spatial localization, as shown in our results of the spatial difference.

**Figure 7:**
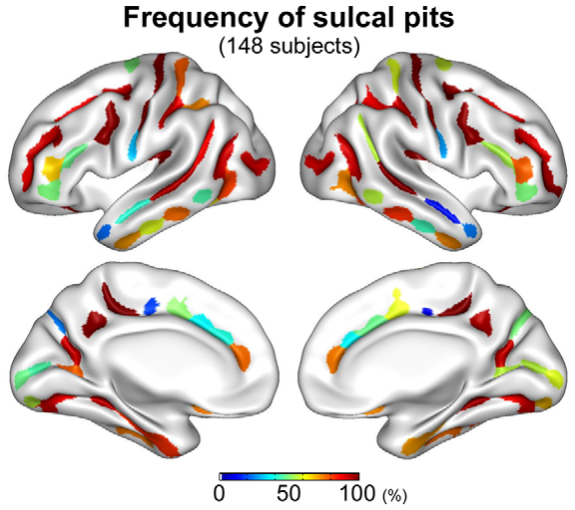
The map of the 96 cluster regions (48 regions for the left and right hemispheres) and the frequency of sulcal pits from the distribution of the pits in 148 normal subjects. This is reproduced from our previous study [2]. The frequency of sulcal pits is represented as a percentage (number of pits/148×100).

We have published a group map of sulcal pits constructed from 148 normal adult brains and defined 96 cluster regions from the distribution of the pits in the group [2]. The map showing the cluster regions and the frequency of sulcal pits as a percentage is reproduced from our previous study (Fig. 7). For further understanding of sulcal pit identification, we visually compared and related the intra-subject reproducibility of the pits with the group map of the sulcal pit frequency. It is of interest that intra-subject reproducibility of sulcal pit extraction seems relatively low in the cingulate sulcal region compared to other regions, and the frequency of sulcal pits in the group is also low (Fig. 6 and 7). It may be because the cingulate sulcus is shallow and its depth profile is not dynamic, increasing uncertainty in identifying sulcal pit location, and also variable across subjects. In the inferior frontal and inferior temporal sulcal regions, low frequency of sulcal pits is shown in the group, but we can see highly reproducible identification of sulcal pits in individual subjects (Fig. 6 and 7). We suggest that low frequency in those regions in the group is not due to unreliable sulcal pit extraction, but due to high inter-subject variability of sulcal patterns.

In conclusion, the extraction of deep sulcal pits and their distribution appears to be highly reliable across scan session, scanner type and extraction tool. The reliability of sulcal pit extraction in shallow regions with small catchment basins, although still high across scan session, scanner type and extraction tool, is less reliable. It may be necessary for us to consider the appropriate way of analysis and interpretation of results for the sulcal pits in those regions. With the high reliability and reproducibility of sulcal pits according to different scan sessions, scanners, and surface extraction pipelines, we suggest that extraction of sulcal pits could provide stable landmarks for studying structure-functional relationship in the human brain, and could be useful for various clinical application studies as an imaging biomarker. Recently multicenter or longitudinal neuroimaging studies, such as the Alzheimer's Disease Neuroimaging Initiative [38], [39], are increasingly becoming an important element of clinical research for diagnosing and evaluating neurological impairments. One of the challenges is to understand and minimize image variability caused by non disease-related factors. Our results suggest that sulcal pits may be useful in multi-center or longitudinal studies, and also comparison between results of cross-sectional studies performed with different image processing pipelines. In future work, it would be important to investigate other instrument-related effects on sulcal pit extraction, such as scanner manufacturer, field strength, or pulse sequence.

## Supporting Information

Figure S1 **Intra-subject cortical surface alignments for 4 subjects.** Eight white matter surfaces are overlapped on a reference volume image.
(TIF)

Figure S2
**An example for measuring similarity of the presence of sulcal pits and their spatial difference (** ***D*** **: spatial difference,** ***G*** **: geodesic distance,** ***N*** **: the number of sulcal pits,** ***P*** **: sulcal pit,** ***S*** **: white matter surface).** Given a pair of surfaces, *SA* and *SB*, containing sulcal pits *PA*  =  {*a1*, *a2*, *a3*} and PB  =  {*b1*, *b2*} respectively, the *a1*, *a2*, and *a3* are projected onto the *S_B_* with the nearest Euclidean distance (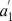,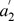 and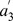) and then the geodesic distances are measured from the *P_B_* on the *S_B_*. The
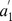
and
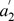
are matched with *b1* and *b2* respectively. The spatial difference is calculated as a mean value of their geodesic distances *G*(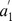, *b1*) and *G*(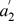,*b2*). We compute the ratio of the number of matched pits (*N*(*P_A→B_*)  =  2) to the whole number of sulcal pits (*N*(*PA*)  =  3). Next, the *b1* and *b2* are projected onto the *SA* and the same measurements are computed. We finally measure the similarity of the presence of sulcal pits and their spatial differences between *SA* and *SB* as shown in figure.(TIF)

Figure S3 **Sulcal pit map for each subject representing the frequency of sulcal pits across the 3 MNI and 3 FS surfaces.** All sulcal pits from different surfaces are projected onto one surface, and the frequency of sulcal pits overlaid in the same region is mapped with color.
(TIF)
