# Supplementary figures and images for: Reliable Identification of Deep Sulcal Pits: The Effects of Scan Session, Scanner, and Surface Extraction Tool
Source: PLoS One. 2013 Jan 7;8(1):e53678. doi: 10.1371/journal.pone.0053678 (PMC3538732; doi:10.1371/journal.pone.0053678)

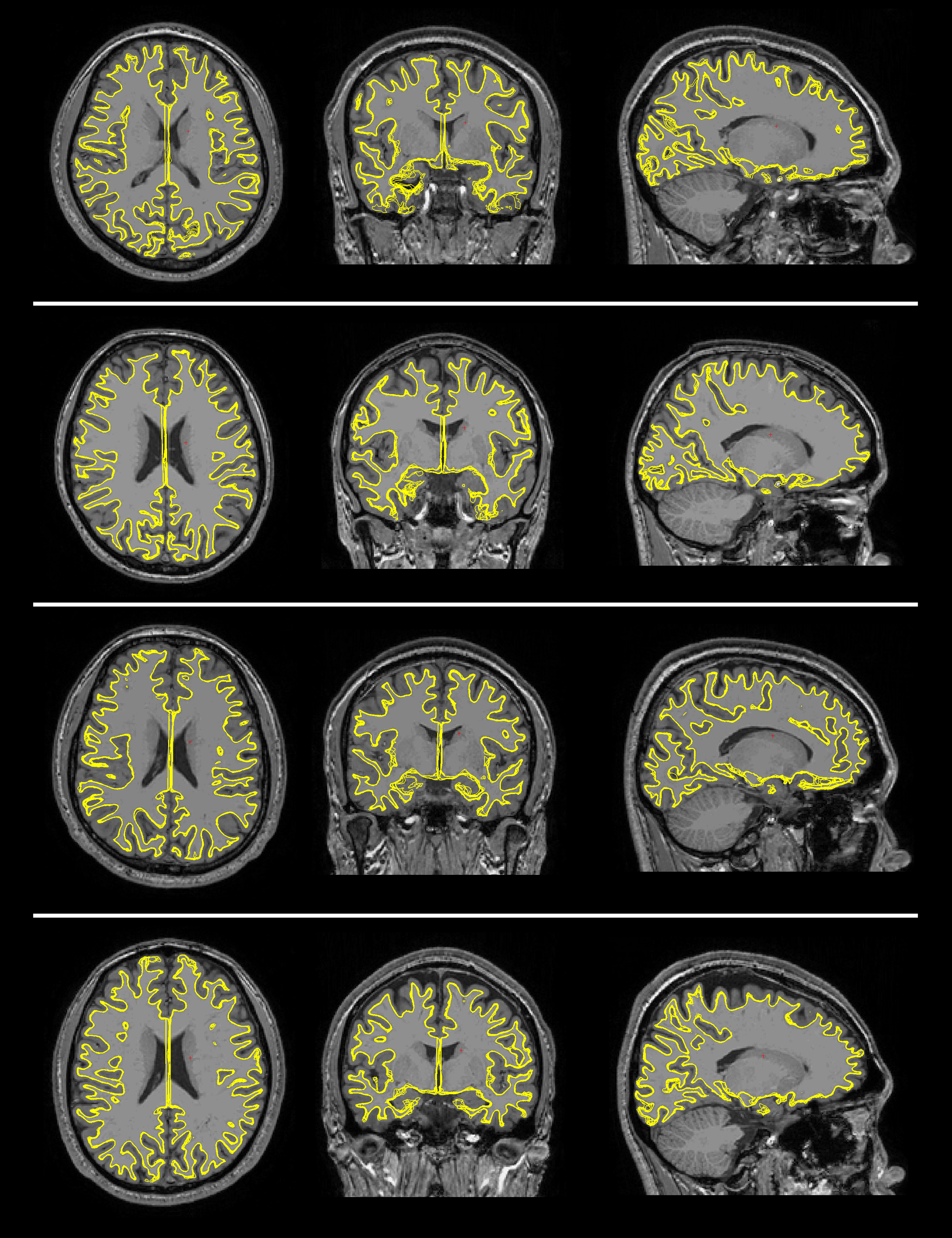

Supplement: Figure S1 — Intra-subject cortical surface alignments for 4 subjects. Eight white matter surfaces are overlapped on a reference volume image. (TIF) [file pone.0053678.s001.tif]

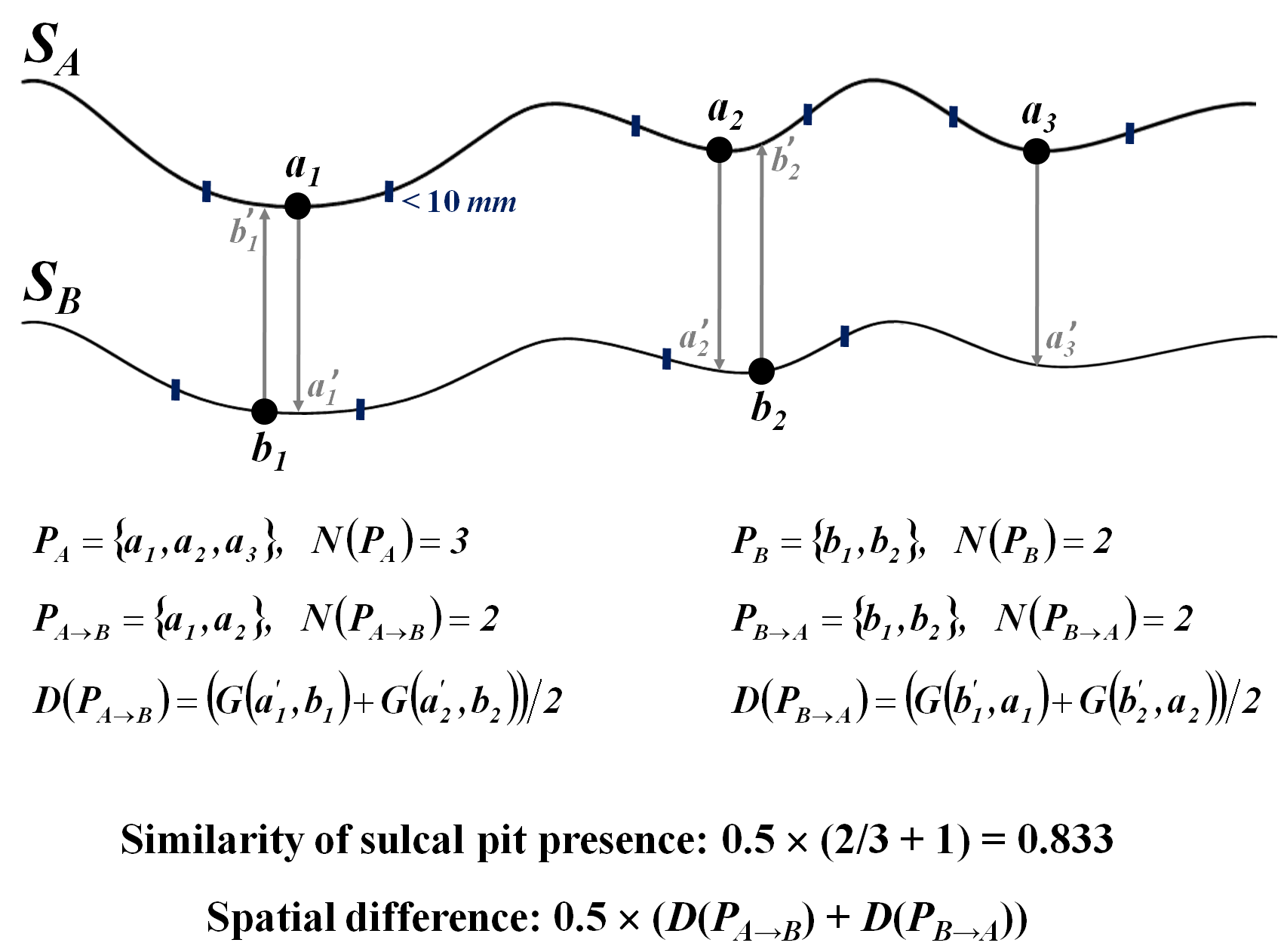

Supplement: Figure S2 — An example for measuring similarity of the presence of sulcal pits and their spatial difference ( D : spatial difference, G : geodesic distance, N : the number of sulcal pits, P : sulcal pit, S : white matter surface). Given a pair of surfaces, SA and SB, containing sulcal pits PA = {a1, a2, a3} and PB = {b1, b2} respectively, the a1, a2, and a3 are projected onto the SB with the nearest Euclidean distance (, and) and then the geodesic distances are measured from the PB on the SB. Theandare matched with b1 and b2 respectively. The spatial difference is calculated as a mean value of their geodesic distances G(, b1) and G(,b2). We compute the ratio of the number of matched pits (N(PA→B) = 2) to the whole number of sulcal pits (N(PA) = 3). Next, the b1 and b2 are projected onto the SA and the same measurements are computed. We finally measure the similarity of the presence of sulcal pits and their spatial differences between SA and SB as shown in figure. (TIF) [file pone.0053678.s002.tif]

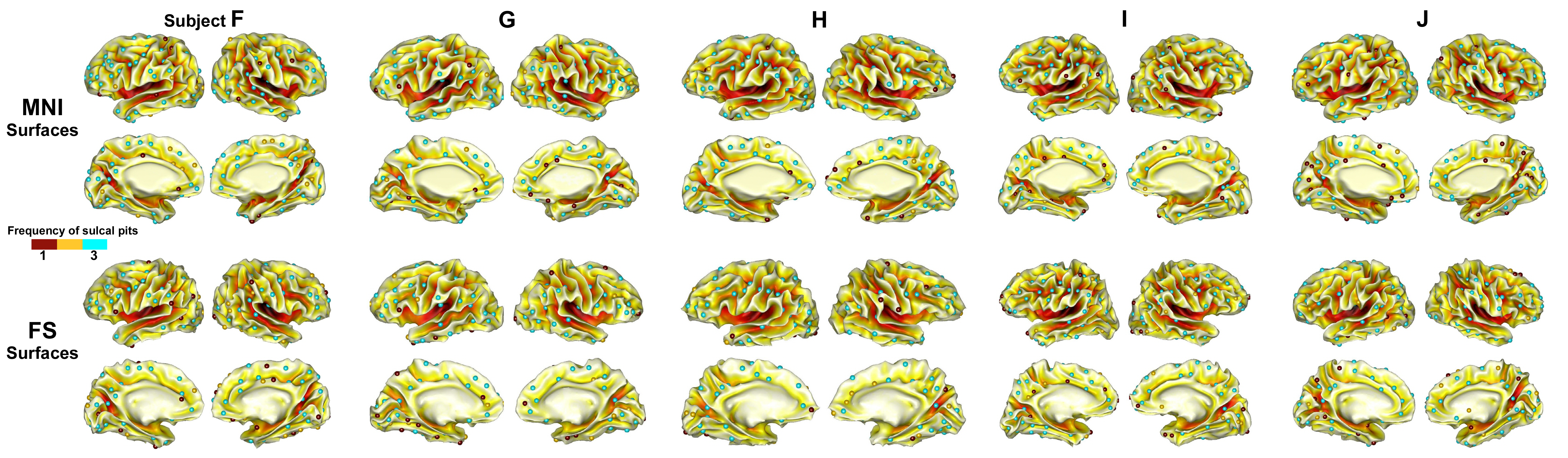

Supplement: Figure S3 — Sulcal pit map for each subject representing the frequency of sulcal pits across the 3 MNI and 3 FS surfaces. All sulcal pits from different surfaces are projected onto one surface, and the frequency of sulcal pits overlaid in the same region is mapped with color. (TIF) [file pone.0053678.s003.tif]
